# Clinicopathological prognostic stratification for proteinuria and kidney survival in IgA nephropathy: a Japanese prospective cohort study

**DOI:** 10.1093/ckj/sfad294

**Published:** 2023-11-27

**Authors:** Kentaro Koike, Tetsuya Kawamura, Keita Hirano, Masako Nishikawa, Akira Shimizu, Kensuke Joh, Ritsuko Katafuchi, Akinori Hashiguchi, Yuichiro Yano, Keiichi Matsuzaki, Masato Matsushima, Nobuo Tsuboi, Shoichi Maruyama, Ichiei Narita, Takashi Yokoo, Yusuke Suzuki

**Affiliations:** Division of Nephrology and Hypertension, Department of Internal Medicine, Jikei University School of Medicine, Tokyo, Japan; Division of Nephrology and Hypertension, Department of Internal Medicine, Jikei University School of Medicine, Tokyo, Japan; Division of Nephrology and Hypertension, Department of Internal Medicine, Jikei University School of Medicine, Tokyo, Japan; Division of Nephrology, Department of Internal Medicine, Japanese Red Cross Ashikaga Hospital, Ashikaga, Japan; Clinical Research Support Center, Jikei University School of Medicine, Tokyo, Japan; Department of Analytic Human Pathology, Nippon Medical School, Tokyo, Japan; Department of Pathology, Jikei University School of Medicine, Tokyo, Japan; National Hospital Organization Fukuoka-Higashi Medical Center, Fukuoka, Japan; Department of Pathology, Keio University School of Medicine, Tokyo, Japan; Department of Advanced Epidemiology, Noncommunicable Disease Epidemiology Research Center, Shiga University of Medical Science, Shiga, Japan; Department of Family Medicine and Community Health, Duke University, Durham, NC, USA; Kyoto University Health Service, Kyoto, Japan; Division of Clinical Epidemiology, Research Center for Medical Sciences, Jikei University School of Medicine, Tokyo, Japan; Division of Nephrology and Hypertension, Department of Internal Medicine, Jikei University School of Medicine, Tokyo, Japan; Department of Nephrology, Nagoya University Graduate School of Medicine, Nagoya, Japan; Niigata University Graduate School of Medical and Dental Sciences, Niigata, Japan; Division of Nephrology and Hypertension, Department of Internal Medicine, Jikei University School of Medicine, Tokyo, Japan; Department of Nephrology, Juntendo University Faculty of Medicine, Tokyo, Japan

**Keywords:** clinicopathological grading, haematuria, IgA nephropathy, kidney prognosis, proteinuria

## Abstract

**Background:**

We require a clinicopathological risk stratification method for immunoglobulin A nephropathy (IgAN) to predict kidney outcomes. We examined a renal failure risk group (RF-RG) classification system created following a prior multicentre, retrospective study to determine if RF-RG could predict kidney outcomes.

**Methods:**

We collected data from Japanese patients with IgAN registered between 1 April 2005 and 31 August 2015. The primary outcome was a composite 50% increase in serum creatinine from baseline or dialysis induction. The secondary outcomes were times to proteinuria remission (ProR) and haematuria remission (HemR).

**Results:**

The enrolled 991 patients from 44 facilities were followed for a median of 5.5 years (interquartile range 2.5–7.5), during which 87 composite events (8.8%) occurred. RF-RG was significantly associated with the primary outcome {hazard ratio [HR] II 2.78 [95% confidence interval (CI) 1.12–6.93], III 7.15 (2.90–17.6), IV 33.4 (14.1–79.0), I as a reference, *P* < .001}.

The discrimination performance was good [C-statistic 0.81 (95% CI 0.76–0.86)] and the time-dependent C-statistics exceeded 0.8 over 10 years. Among the 764 patients with proteinuria and 879 patients with haematuria at baseline, 515 and 645 patients showed ProR and HemR, respectively. ProR was significantly less frequent in patients with advanced disease [subdistribution HR: II 0.79 (95% CI 0.67–0.94), III 0.53 (0.41–0.66), IV 0.15 (0.09–0.23), I as a reference, *P* < .001]. We also observed an association between HemR and RF-RG.

**Conclusions:**

RF-RG demonstrated good predictive ability for kidney outcomes.

## Graphical Abstract

**Graphical Abstract:**
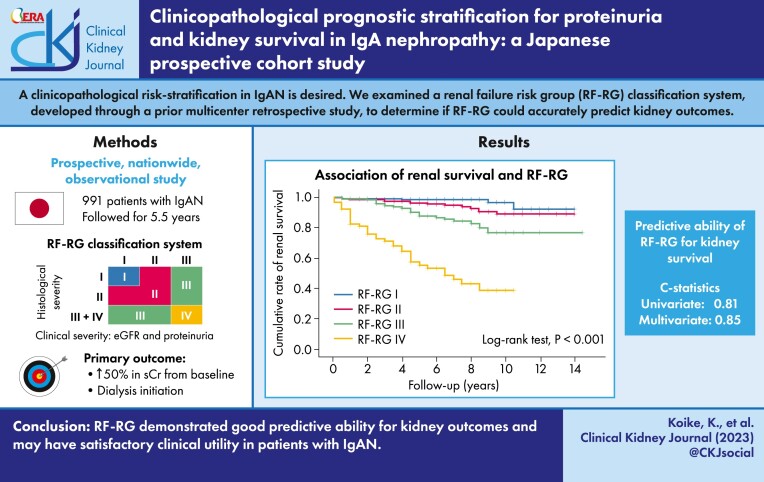

KEY LEARNING POINTS **What was known:**

- The International IgAN Prediction Tool, published in 2019, uses various parameters obtained at the time of kidney biopsy in immunoglobulin A nephropathy (IgAN) to calculate the risk of kidney dysfunction. The International IgAN Prediction Tool has been reported to have good prognostic ability in several validation studies.
- In Japan, the renal failure risk group (RF-RG), which incorporated histological and clinical severity, has been reported and widely used. However, the validity of the RF-RG as a prognostic classification has never been evaluated in an external cohort.

**This study adds:**

- This study analysed the effectiveness of the RF-RG in predicting kidney prognosis in IgAN by utilizing the data from the Japan IgA nephropathy prospective cohort study. The results revealed a significant association between the RF-RG and kidney prognosis.
- This study demonstrated that the RF-RG is a reliable predictor of kidney outcomes with good predictive capacity.

**Potential impact:**

- The RF-RG can efficiently stratify the risk of kidney dysfunction by using pathological findings of kidney biopsy, estimated glomerular filtration rate and proteinuria. It can be easily incorporated into the routine practice of IgAN and has the potential to become widely used alongside the International IgAN Prediction Tool.

## INTRODUCTION

Immunoglobulin A nephropathy (IgAN) is the most prevalent glomerulonephritis worldwide. Approximately 40% of patients with IgAN eventually progress to end-stage kidney disease (ESKD) within 20 years [1]. Prognostication incorporates clinical and pathological information to predict kidney outcomes; however, only a few clinicopathological prediction tools are available for IgAN [2]. In 2019, the International IgAN Network performed a retrospective multinational study to establish a risk prediction scale based on clinicopathological data at kidney biopsy [3]. The model showed good discrimination in an external validation cohort (Harrell's C-statistic 0.82). The model's formula uses MEST-C scores of the Oxford classification with a split system; mesangial hypercellularity (M; M0/1), endocapillary hypercellularity (E; E0/1), segmental sclerosis (S; S0/1), interstitial fibrosis/tubular atrophy (T; T0/1/2) and crescent (C; C0/1/2). The authors found no significant associations between each MEST-C score and kidney survival, except for the T score.

Susceptibility for IgAN is higher in East Asian countries than in the USA and Europe [4, 5]. The International IgAN Network found a 1.5- to 2.0-fold increased risk of kidney events in Japanese and Chinese patients compared with their Caucasian counterparts [3]. The Japanese IgAN Study Group started a nationwide prospective multicentre IgAN cohort study [Japan IgAN Prospective Cohort Study (J-IGACS)] on 1 April 2005. This study sought to elucidate the relationship between clinical and pathological findings at kidney biopsy and long-term kidney outcomes. A simultaneous retrospective multicentre cohort study created an evidence-based grading system for kidney survival in 2005 and defined clinicopathological prognostic grades represented by ESKD risk groups (RGs) I–IV in 2011. These grades incorporated clinical grade (CG; I–III) and histological grade (HG; I–IV) [6, 7]. To clarify that RG refers to the ESKD risk group, this article uses the term renal failure risk group (RF-RG) instead of RG as used in previous studies. While HG was previously validated on an external cohort [8], CG and RF-RG have not been externally validated.

Earlier studies demonstrated an association between proteinuria remission (ProR) and kidney survival; an association between haematuria remission (HemR) and kidney survival was also reported recently [9, 10]. However, no studies have examined these variables in the same cohort. Although urinary abnormality remission during follow-up may be important for predicting kidney survival, there is no classification model to predict urinary abnormality remission for patients with IgAN.

We evaluated the predictive performance of the RF-RG on kidney survival, incorporating CG and HG, and applied these stratifications to patients experiencing urinary abnormality remission. Simultaneously, we examined potential associations between remission of urinary abnormality and kidney survival within the J-IGACS.

## MATERIALS AND METHODS

### Study population

Patients with biopsy-proven primary IgAN from Japanese renal units were enrolled in J-IGACS from 1 April 2005 to 31 August 2015. The inclusion criteria were a new diagnosis of IgAN by kidney biopsy and kidney biopsy specimen(s) containing ≥10 glomeruli [11]. Informed consent was obtained from each patient to use his/her clinical data and all study procedures followed the Declaration of Helsinki. The Jikei University School of Medicine's Institutional Review Board on Human Research approved this research [no. 16-174 (4402)], as did each participating institute's local ethics committee. The study's results are reported following the Strengthening the Reporting of Observational Studies in Epidemiology guidelines [12].

### Data collection

We collected patients’ age, sex, mean arterial pressure (MAP), serum creatinine (SCr), estimated glomerular filtration rate (eGFR), uric acid (UA), urinary protein excretion rate (UPER) and red blood cells within the urinary sediment (URBC) at the time of the kidney biopsy and during follow-up. For patients <20 years of age at the time of their kidney biopsy, eGFR was calculated using Uemura's equation; follow-up eGFR measurements were calculated once the patient was >20 years of age using Matsuo's equation [13, 14]. Clinical datasets were uploaded every 6 months to the J-IGACS website, maintained by the University Hospital Medical Information Network. We defined the treatments initiated up to 1 year after a kidney biopsy as initial treatments. Initial treatments included corticosteroids (CS2 for regimens with pulses, CS1 for regimens without pulses and CS0 for those without corticosteroid therapy), tonsillectomy (Tx1 performed and Tx0 unperformed) and renin–angiotensin system inhibitors (RASis). Regarding RASis, if the patients had already received an RASi at their kidney biopsy, such therapies were recorded as an initial treatment. All patients were followed up until 31 May 2021.

### Study protocol revision

We initially planned to observe each study patient for >10 years after enrolment, meaning follow-up would end in August 2025. Because more patients would receive additional treatment, this protracted follow-up could affect the validity of the grading at the time of biopsy and initial treatment effectiveness. Therefore, the study protocol was revised to shorten the follow-up period. We also changed the primary outcome from a 100% increase in serum creatinine (SCr) to a 50% increase.

### Clinicopathological prognostic stratification for IgAN

**Figure 1:**
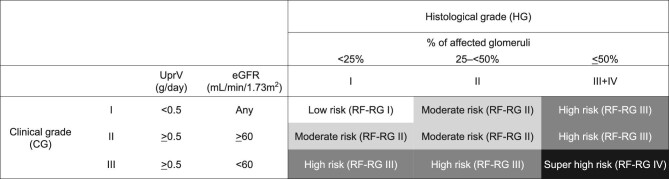
Chart of the ESKD risk group produced by combining clinical and histological grades. This chart was modified from our previous studies [15, 16]. CG I, II and III consisted of those patients with proteinuria <0.5 g/day, those with proteinuria ≥0.5 g/day and eGFR ≥60 ml/min/1.73 m^2^ and those with proteinuria ≥0.5 g/day and eGFR <60 ml/min/1.73 m^2^, respectively. HG I, II, III and IV were established corresponding to <25%, 25–<50%, 50–<75% and 75–100% of glomeruli exhibiting cellular or fibrocellular crescents, global sclerosis, segmental sclerosis or fibrous crescents, respectively. Our previous retrospective study classifies the RF-RG into four levels according to the odds ratio of the individual compartments defined by combining CG and HG [15].

RF-RG I–IV combined CG I–III and HG I–IV and were created based on the results of prior studies (Fig. 1) [6, 15]. CG was classified into three levels based on the UPER and eGFR (CG I: UPER <0.5 g/day; CG II: UPER ≥0.5 g/day and eGFR ≥60 ml/min/1.73 m^2^; CG III: UPER ≥0.5 g/day and eGFR <60 ml/min/1.73 m^2^). The four HG levels were HG I: <25%, HG II: 25–<50%, HG III: 50–<75% and HG IV: ≥75% of total glomeruli exhibiting cellular/fibrocellular/fibrous crescents and segmental/global sclerosis [16]. Biopsy specimens were collected and converted into virtual images. Five pathologists blinded to the patients’ clinical data analysed the same virtual images. In this study, they rated individual biopsy findings as HG I–IV. The HG level was determined according to the consensus of three or more kidney pathologists.

### Outcomes

We defined the primary outcome as a composite 50% increase in SCr from baseline or dialysis induction. This variable was adopted by several prior randomized controlled trials for IgAN as the primary endpoint [17, 18]. In patients <20 years of age at the time of their kidney biopsy, the primary outcome was a composite kidney outcome of dialysis induction or a 25% decrease in eGFR from its baseline value.

The secondary outcomes included ProR and HemR, defined as proteinuria of <0.3 g/day or 0.3 g/g Cr and URBC of <5/high power field for two consecutive measurements over at least 6 months before primary outcome occurrence [19].

An exploratory analysis was also performed using a decrease in eGFR (30% or 50%) from its baseline value or dialysis induction as an alternative outcome.

### Analysis sets

Dataset 1 was used for the primary analysis. We excluded patients missing one or more baseline eGFR, UPER, HG or URBC measures or those without follow-up data. Dataset 2 and dataset 3 were used for the analysis of ProR and HemR, respectively. We excluded patients who satisfied the definitions of ProR or HemR at baseline from dataset 2 and dataset 3, respectively. Dataset 4 was used to analyse the association between kidney survival and remission of urine abnormalities; here, we excluded patients who satisfied the definitions of ProR or HemR at baseline.

### Statistical analysis

Patients’ baseline characteristics are presented as mean values with standard deviations (SDs) or medians with interquartile ranges (IQRs) for continuous variables and percentages for categorical variables. According to the median URBC, patients were classified into two groups based on microscopic haematuria severity. Single imputation was performed to address missing baseline MAP and UA data. We assumed a multivariate normal distribution by sex for UA, MAP, age, eGFR and log-transformed UPER.

The time from origin to the occurrence of primary and secondary outcomes was defined as the time of the patient's kidney biopsy. The event date was the first day of two consecutive laboratory examinations where their respective criteria were met. The time-to-event data were treated as censored at the latest respective examination date if the events were unconfirmed. Cumulative kidney survival was determined using the Kaplan–Meier method. Logrank tests were used to compare primary outcomes among the various RF-RG, CG and HG levels.

Since patients transferred to dialysis do not achieve urinary remission, and because a 50% increase in SCr was considered a surrogate for dialysis, we treated the primary outcome as a competing risk for respective secondary outcomes in the analyses. If the follow-up record durations differed between the primary and secondary outcomes in the same patient, we truncated the longer time-to-event data using the shorter duration for the competing risk analyses. The cumulative incidence of secondary outcomes was estimated using competing risk methodology by the cumulative incidence function (CIF) [20]. We used Gray's test to compare the secondary outcomes among the various levels of RF-RG, CG and HG [21].

To explore the predictive accuracy of the grading systems for the primary outcome, we used the univariate Cox regression models with Akaike information criteria (AIC) and the C-statistic. We calculated the time-dependent C-statistic [the area under the time-dependent receiver operating characteristic curve (time-dependent AUC)] by the univariate and multivariate Cox regression models using Uno *et al.*’s method [22]. Subgroup analyses by baseline characteristics—including age, MAP, URBC and sex—and initial treatment—including RASi, CS and Tx—were also performed as a sensitivity analysis using dataset 1, where patients were divided into two subgroups based on median values for continuous or categorical variables. To explore the association of the grading systems for the secondary outcomes, we used the univariate and multivariate Fine–Gray models with AIC [23]. To explore the influence of secondary outcomes on the primary outcome, multivariate Cox regression with both time-dependent remissions statuses (when and whether secondary outcomes were achieved) as explanatory variables were compared using the same Cox regression without the time-dependent explanatory variables by AIC. All statistical tests were two-sided and *P*-values <.05 were considered statistically significant in all analyses. We performed all analyses using JMP 13.2.0 (JMP Statistical Discovery, Cary, NC, USA), SAS 9.4 (SAS Institute, Cary, NC, USA), SPSS 25 (IBM, Armonk, NY, USA) and Stata/MP 16.1 (StataCorp, College Station, TX, USA) statistical software programs.

## RESULTS

### Baseline characteristics and follow-up

**Figure 2:**
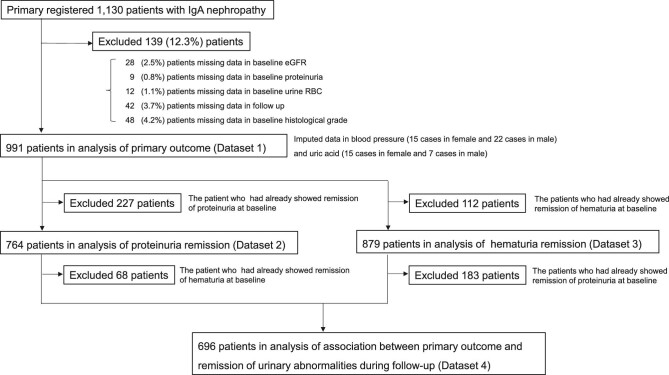
Patient flow.

Based on the inclusion criteria, data were collected from 1130 patients with IgAN registered at 44 Japanese centres (Supplementary Table S1); 139 were excluded and 991 were analysed for the primary outcome (dataset 1 in Fig. 2). The mean values for eGFR and UPER were 75.4 ml/min (SD 8.7) and 0.58 g/day (IQR 0.28–10.18), respectively. Corticosteroids were administered to 64% (634/991) and 42.9% (425/991) of patients underwent tonsillectomy. Patient characteristics by RF-RG are shown in Table 1. As the RF-RG severity level increased, age, MAP and UPER increased and eGFR decreased. A similar trend was observed for CG and HG (Supplementary Table S2).

**Table 1:** Baseline characteristics and follow-up period in 991 patients analysed for primary outcome (dataset 1).

		RF-RG			
Characteristics	Overall (*N* = 991)	I (*n* = 358)	II (*n* = 377)	III (*n* = 165)	IV (*n* = 91)
At baseline					
Age (years), median (IQR)	37.1 (26.8–50.4)	31.1 (22.1–41.9)	35.7 (26.4–46.8)	48.6 (38.1–60.8)	49.2 (36.1–63.5)
Female, *n* (%)	502 (50.7)	190 (53.1)	195 (51.7)	80 (48.5)	37 (40.7)
MAP (mmHg), mean ± SD	90.1 ± 13.5	85.6 ± 11.5	89.6 ± 13.2	94.7 ± 12.0	102.0 ± 15.1
eGFR (ml/min/1.73 m^2^), mean ± SD	75.4 ± 28.7	87.9 ± 24.0	83.8 ± 23.6	52.3 ± 17.4	32.9 ± 14.7
UPER (g/day), median (IQR)	0.58 (0.28–1.18)	0.23 (0.10–0.34)	0.80 (0.55–1.23)	1.23 (0.72–2.00)	2.11 (1.08–3.50)
URBC >20/hpf, *n* (%)	514 (51.9)	188 (52.5)	190 (50.4)	97 (58.8)	39 (42.9)
UA (mg/dl), mean ± SD	5.9 ± 1.6	5.4 ± 1.5	5.6 ± 1.4	6.7 ± 1.5	7.5 ± 1.7
RASi, *n* (%)	567 (57.2)	126 (35.2)	226 (59.9)	133 (80.6)	82 (90.1)
CS0/CS1/CS2, *n* (%)	357 (36.0)/57 (5.8)/577 (58.2)	175 (48.9)/17 (4.7)/166 (46.4)	98 (26.0)/21 (5.6)/258 (68.4)	48 (29.1)/12 (7.3)/105 (63.6)	36 (39.6)/7 (7.7)/48 (52.7)
Tonsillectomy, *n* (%)	425 (42.9)	155 (43.3)	179 (47.5)	63 (38.2)	28 (30.8)
Follow-up					
Follow-up period (months), median (IQR)	66 (30–90)	60 (24–90)	72 (36–96)	72 (36–96)	36 (12–72)

hpf: high-power field.

### Association between RF-RG and primary outcome

**Figure 3:**
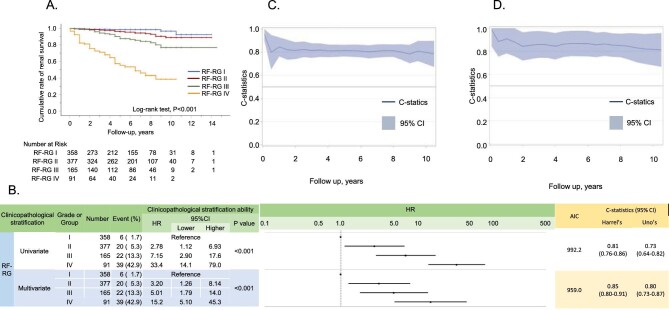
The association between the primary outcome and RF-RG. **(A)** Kaplan–Meier survival curves showed significantly different kidney outcomes relative to the RF-RG level. **(B)** Univariate and multivariate Cox regression models (upper panel: univariate; lower panel: multivariate) revealed a significant association for the primary outcome by the RF-RG. The multivariate models were adjusted for age, sex, MAP, eGFR, UPER, URBC, UA and initial treatment with RASis, glucocorticoids and tonsillectomy. Both univariate and multivariate models show good discrimination, as indicated by high C-statistic values. The time-dependent AUC in univariate and multivariate analyses by the RF-RG revealed good discriminatory performance of the RF-RG for the primary outcome over 10 years in **(C)** univariate and **(D)** multivariate analyses.

During the median follow-up of 5.5 years, 87 primary outcomes (8.8%) occurred: 6 (1.7%) in RF-RG I, 20 (5.3%) in RF-RG II, 22 (13.3%) in RF-RG III and 39 (42.9%) in RF-RG IV. The RF-RG was significantly associated with the primary outcome, as shown by Kaplan–Meier survival curves (Fig. 3A). Likewise, univariate Cox regression analyses (Fig. 3B) showed significant associations between the RF-RG and the primary outcome. The risk of the primary outcome increased as the RF-RG level increased. The discriminatory performance of the RF-RG for the primary outcome was good (Harrell's C-statistic >0.8). These key findings were ascertained by multivariate analyses (Fig. 3B), adjusted by the baseline characteristics and initial treatments described in Supplementary Table S3. Moreover, the time-dependent C-statistics exceeded 0.8 over 10 years during univariate and multivariate analyses for the primary outcome (Fig. 3C, D). Subgroup analyses (Table 2) also demonstrated a significant association between the RF-RG and primary outcome, independent of baseline characteristics and initial treatments.

**Table 2:** Comparison of primary outcome among RF-RG I–IV in subgroups according to baseline characteristics.

Subgroup		RF-RG	*n*	Events, *n* (%)	HR	95% CI	Wald's *P*-value	*P* int.
Age	Younger	I	234	3 (1.3)	Reference		<.001	.69
		II	205	9 (4.4)	5.44	1.35–22		
		III	31	3 (9.7)	17.5	2.37–129.2		
		IV	26	10 (38.5)	174.9	17.7–1728.6		
	Older	I	123	2 (1.8)	Reference		.018	
		II	172	11 (3.4)	3.59	0.98–13.2		
		III	134	19 (12.9)	3.37	0.93–12.2		
		IV	65	29 (43.1)	7.03	1.87–26.4		
Sex	Female	I	190	4 (2.1)	Reference		.001	.74
		II	195	8 (4.1)	2.33	0.64–8.41		
		III	80	10 (12.5)	6.68	1.48–30.1		
		IV	37	14 (38.7)	30.9	5.40–177.4		
	Male	I	168	2 (1.2)	Reference		.015	
		II	182	12 (6.6)	5.832	1.27–26.769		
		III	85	12 (14.1)	5.807	1.191–28.315		
		IV	54	25 (46.3)	12.18	2.43–61.05		
MAP	Lower	I	251	3 (1.2)	Reference		.003	.14
		II	219	11 (5.0)	5.45	1.42–20.9		
		III	64	8 (12.5)	13.1	2.69–63.6		
		IV	23	6 (26.1)	32.2	5.04–206.0		
	Higher	I	107	3 (2.8)	Reference		.005	
		II	158	9 (5.7)	2.06	0.55–7.73		
		III	101	14 (13.9)	2.66	0.69–10.3		
		IV	68	33 (48.5)	8.03	1.95–33.1		
URBC	Lower	I	170	3 ( 1.8)	Reference		.001	.35
		II	187	12 ( 6.4)	3.22	0.88–11.8		
		III	68	11 (16.2)	5.35	1.25–22.9		
		IV	52	29 (55.8)	15.8	3.52–70.8		
	Higher	I	188	3 (1.6)	Reference		.002	
		II	190	8 (4.2)	3.40	0.86–13.5		
		III	97	11 (11.3)	4.33	0.95–19.6		
		IV	39	10 (25.6)	27.1	4.44–165.7		
RASi	No	I	232	3 (1.3)	Reference		.039	.85
		II	151	5 (3.3)	1.89	0.34–10.7		
		III	32	2 (6.3)	6.39	0.36–113.6		
		IV	9	2 (22.2)	134.2	4.08–4413.5		
	Yes	I	126	3 (2.4)	Reference		<.001	
		II	226	15 (6.6)	4.07	1.16–14.3		
		III	133	20 (15.0)	4.98	1.40–17.7		
		IV	82	37 (45.1)	13.2	3.56–48.7		
CS	No	I	175	3 (1.7)	Reference		.002	.48
		II	98	10 (10.2)	4.26	1.13–16.0		
		III	48	15 (31.3)	9.83	2.34–41.2		
		IV	36	22 (61.1)	21.3	4.37–103.9		
	Yes	I	183	3 (1.6)	Reference		.004	
		II	279	10 (3.6)	1.90	0.50–7.27		
		III	117	6 (6.0)	1.32	0.27–6.37		
		IV	55	17 (30.9)	7.74	1.56–38.4		
Tonsillectomy	No	I	203	4 (2.1)	Reference		<.001	.45
		II	198	9 (5.0)	2.85	1.00–8.10		
		III	102	17 (18.1)	4.35	1.42–13.3		
		IV	63	29 (52.7)	13.6	4.02–46.2		
	Yes	I	155	1 (0.6)	Reference		.06	
		II	179	6 (3.4)	3.81	0.41–35.1		
		III	63	3 (4.8)	6.19	0.41–93.7		
		IV	28	5 (17.9)	30.1	1.88–484.8		

Cut-off values of age, MAP, eGFR, proteinuria, URBC was 38 years old, 90 mmHg, 21/high-power field, respectively.

The association between the RF-RG and kidney prognosis was also confirmed in exploratory analysis using a 30% and 50% decrease in eGFR from the baseline value as an outcome (Supplementary Fig. S1, Supplementary Table S4).

### Association between RF-RG and remission of urinary abnormalities

The characteristics of the patients analysed for ProR (dataset 2 in Fig. 2, *n* = 764) and HemR (dataset 3 in Fig. 2, *n* = 879) are summarized in Tables 3 and 4, respectively. Among patients with overt proteinuria at baseline, 515 (67.4%) experienced remission over a median follow-up of 1.0 years. Likewise, remission was observed in 645 (73.4%) among those with haematuria at baseline over a median follow-up of 1.5 years.

**Table 3:** Baseline characteristics and follow-up period in 764 patients analysed for proteinuria remission (dataset 2).

		RF-RG			
Characteristics	Overall (*N* = 764)	Ⅰ (*n* = 162)	Ⅱ (*n* = 353)	Ⅲ (*n* = 160)	Ⅳ (*n* = 89)
At baseline					
Age (years), median (IQR)	38.7 (28.1–51.6)	33.2 (21.8–42.4)	35.5 (26.2–46.2)	48.4 (38.2–61.1)	49.2 (36.0–63.8)
Female, *n* (%)	377 (49.3)	80 (49.4)	182 (51.6)	78 (48.8)	37 (41.6)
MAP (mmHg), mean ± SD	91.1 ± 13.7	85.3 ± 11.7	89.6 ± 13.3	94.7 ± 12.1	101.3 ± 14.5
eGFR (ml/min/1.73 m^2^), mean ± SD	72.7 ± 29.0	88.2 ± 23.3	84.7 ± 23.5	52.3 ± 12.1	33.5 ± 14.3
UPER (g/day), median (IQR)	0.80 (0.46–1.41)	0.35 (0.27–0.43)	0.84 (0.58–1.27)	1.28 (0.73–2.03)	2.07 (1.07–3.49)
URBC >20/hpf, *n* (%)	401 (52.5)	87 (53.7)	179 (50.7)	96 (60.0)	39 (43.8)
UA (mg/dl), mean ± SD	6.0 ± 1.7	5.5 ± 1.6	5.5 ± 1.4	6.6 ± 1.5	7.4 ± 1.7
RASi, *n* (%)	487 (63.7)	63 (38.9)	212 (60.1)	131 (81.9)	81 (91.0)
CS0/CS1/CS2, *n* (%)	242 (31.7)/46 (6.0)/476 (62.3)	75 (46.3)/7 (4.3)/80 (49.4)	87 (24.6)/20 (5.7)/246 (69.7)	45 (28.1)/12 (7.5)/103 (64.4)	35 (39.3)/7 (7.9)/47 (52.8)
Tonsillectomy, *n* (%)	332 (43.5)	72 (44.4)	170 (48.2)	62 (38.8)	28 (31.5)
Follow-up					
Follow-up period (months), median (IQR)	12 (6–36)	12 (6–18)	12 (6–30)	18 (6–42)	24 (12–48)

hpf: high-power field.

**Table 4:** Baseline characteristics and follow-up period in 879 patients analysed for haematuria remission (dataset 3).

		RF-RG			
Characteristics	Overall (*N* = 879)	Ⅰ (*n* = 309)	Ⅱ (*n* = 340)	Ⅲ (*n* = 152)	Ⅳ (*n* = 78)
At baseline					
Age (years), median (IQR)	36.3 (26.2–49.1)	29.6 (21.1–40.7)	35.2 (26.0–46.2)	48.9 (38.2–60.9)	47.3 (35.7–63.6)
Female, *n* (%)	449 (51.1)	165 (53.4)	177 (52.1)	74 (48.7)	33 (42.3)
MAP (mmHg), mean ± SD	89.7 ± 13.5	85.3 ± 11.9	89.0 ± 13.1	94.6 ± 12.2	100.8 ± 14.7
eGFR (ml/min/1.73 m^2^), mean ± SD	76.3 ± 28.9	89.3 ± 24.0	84.8 ± 23.6	52.6 ± 17.8	33.7 ± 14.1
UPER (g/day), median (IQR)	0.59 (0.29–1.21)	0.24 (0.11–0.34)	0.80 (0.56–1.21)	1.31 (0.73–2.05)	2.12 (1.08–3.76)
URBC >20/hpf, *n* (%)	506 (57.6)	183 (59.2)	188 (55.3)	96 (63.2)	39 (50.0)
UA (mg/dl), mean ± SD	5.8 ± 1.6	5.4 ± 1.5	5.6 ± 1.4	6.7 ± 1.5	7.5 ± 1.7
RASi, *n* (%)	494 (56.2)	102 (33.0)	200 (58.8)	121 (79.6)	71 (91.0)
CS0/CS1/CS2, *n* (%)	297 (33.8)/53 (6.0)/529 (60.2)	152 (49.2)/15 (4.9)/142 (46.0)	77 (22.6)/20 (5.9)/243 (71.5)	40 (26.3)/11 (7.2)/101 (66.4)	28 (35.9)/7 (9.0)/43 (55.1)
Tonsillectomy, *n* (%)	394 (44.8)	136 (44.0)	171 (50.3)	60 (39.5)	27 (34.6)
Follow-up					
Follow-up period (months), median (IQR)	18 (12–36)	18 (12–36)	18 (12–36)	18 (6–30)	12 (10.5–24)

**Figure 4:**
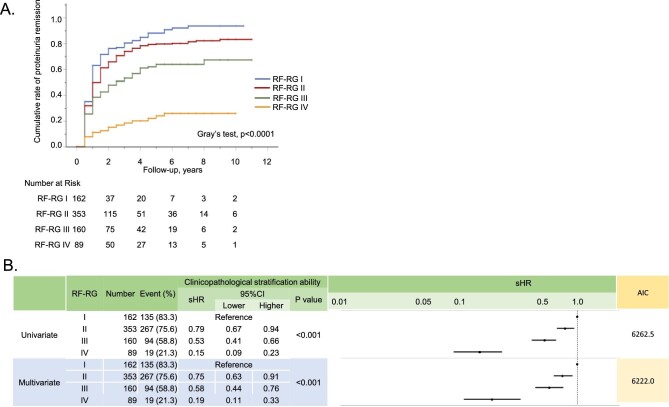
Association between proteinuria remission and the RF-RG. **(A)** The CIF curves and Gray's tests showed significantly different rates of proteinuria remission among RF-RG I–IV. **(B)** The subdistribution hazard ratio (sHR) among RF-RG I–IV for proteinuria remission using the Fine-Gray model (see Supplementary Table S5).

**Figure 5:**
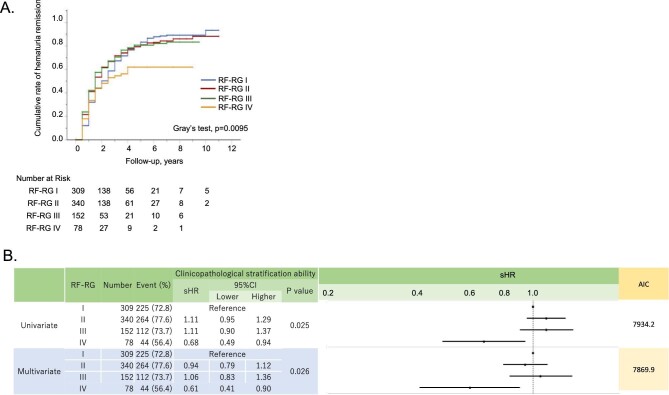
Association between haematuria remission and RF-RG. **(A)** The CIF curve shows that as the RF-RG level increases, the remission rate of haematuria decreases. Gray's tests showed significantly different rates of haematuria remission among RF-RG I–IV. **(B)** The subdistribution hazard ratio (sHR) among RF-RG I–IV for haematuria remission according to the Fine–Gray model (see Supplementary Table S6).

As shown by CIF curves (Fig. 4A) and univariate Fine–Gray regression models (Fig. 4B), ProR was significantly less frequent in higher RF-RG levels. These associations were also ascertained by multivariate models (Fig. 4B, Supplementary Table S5), adjusted for baseline characteristics and initial treatments (Table 1). The CIF curves and Gray's test showed a significant association between HemR and RF-RG levels (Fig. 5, Supplementary Table S6).

### Predictive ability of CG and HG for the primary and secondary outcomes

As with the RF-RG, CG and HG were significantly associated with the primary outcome, as shown by Kaplan–Meier survival curves (Supplementary Figs. S2A, B). Univariate and multivariate Cox regression analyses showed significant associations between CG and HG and the primary outcome. The risk of the primary outcome increased as CG and HG levels increased. Harrell's C-statistics of univariate analysis were 0.75 for CG and 0.82 for HG (Supplementary Fig. S2C, Supplementary Table S7). Our analysis of the association between ProR and CG or HG revealed that ProR was significantly less frequent for patients classified into higher CG and HG levels, as indicated by the CIF curves (Supplementary Figs. S3A, B). These associations were also ascertained by univariate and multivariate Fine–Gray regression models (Supplementary Fig. S3C, Supplementary Table S8). Lastly, HemR was significantly less frequent in higher HG levels (Supplementary Fig. S4, Supplementary Table S9).

### Urinary abnormality remission and the primary outcome

To determine the association between the occurrence of urinary abnormality remission and the primary outcome, we analysed patients included in dataset 4 and depicted in Fig. 2 (*n* = 696) (Supplementary Table S10). Similar to dataset 1, the multivariate model with the factors of urine abnormality remission showed that the RF-RG was significantly associated with the primary outcome. ProR and HemR were significantly associated with the primary outcome in the model. Of note, when we compared model 2, with the factors of urinary abnormality remission, and model 1, without the factors of urinary abnormality remission by AIC, model 2 had a remarkably improved goodness-of-fit and an AIC difference of 44.2 (Table 5).

**Table 5:** Association between primary outcome and urinary remissions in the patients with both proteinuria and haematuria at baseline (dataset 4).

					Multivariate			
					Model 1		Model 2	
		Univariate			AIC	764.3	AIC	720.1
Predictors		HR (95% CI)	*P*-value	AIC	HR (95% CI)	*P*-value	HR (95% CI)	*P*-value
RF-RG	I	Reference	<.001	809.1	Reference	<.001	Reference	.002
	II	4.01 (0.93–17.3)			4.89 (1.12–21.3)		5.72 (1.31–25.0)	
	III	10.4 (2.43–44.3)			8.02 (1.72–37.5)		7.35 (1.57–34.5)	
	IV	44.8 (10.7–187.0)			23.0 (4.63–113.9)		17.6 (3.49–88.5)	
Baseline clinical parameters								
Age, per 10 years		1.53 (1.31–1.78)	<.001	864.8	0.99 (0.81–1.20)	.878	1.05 (0.86–1.27)	.644
Sex (ref male)		0.62 (0.39–0.99)	.046	891.8	0.76 (0.45–1.30)	.317	0.70 (0.41–1.17)	.170
MAP, per 10 mmHg		1.39 (1.20–1.61)	<.001	877.9	1.05 (0.88–1.25)	.612	1.07 (0.90–1.28)	.459
eGFR, per 10 ml/min		0.62 (0.56–0.70)	<.001	816.4	0.89 (0.73–1.07)	.213	0.87 (0.72–1.07)	.184
UPER, per 1.0 g/day		1.08 (1.04–1.11)	<.001	886.1	1.07 (1.02–1.12)	.007	1.07 (1.02–1.13)	.004
URBC >20/hpf, yes versus no		0.48 (0.30–0.77)	.002	886.1	0.66 (0.40–1.09)	.101	0.57 (0.33–0.98)	.042
UA, per 1 mg/dl		1.46 (1.30–1.66)	<.001	865.2	0.99 (0.82–1.21)	.936	1.02 (0.85–1.21)	.865
Urinary abnormality during follow-up^a^								
Proteinuria remission, yes versus no		0.08 (0.04–0.15)	<.001	814.6	ND		0.15 (0.07–0.31)	<.001
Haematuria remission, yes versus no		0.29 (0.17–0.50)	<.001	876.4	ND		0.29 (0.15–0.55)	<.001

hpf: high-power field; ND: not determined.

^a^Remissions status was treated as a time-dependent variable.

Model 1 was adjusted for initial treatment with RASi, glucocorticoid and tonsillectomy in addition to the parameters listed in each column. Model 2 was adjusted with the same variables as in Model 1 and urinary remission (yes or no).

## DISCUSSION

This prospective observational study with IgAN confirmed the good predictive ability of the RF-RG for kidney survival. This study demonstrated that the RF-RG has a good and stable prediction ability for kidney survival as presented by the time-dependent AUC, independent of baseline characteristics. Furthermore, this key message was consistently confirmed in subgroup analyses by baseline characteristics in Table 2. Moreover, the present J-IGACS revealed various distinctive features described below.

Predictive consistency for primary outcome by the RF-RG originated with the HG. The discriminative performances of HG and RF-RG for kidney survival were good. The HG was constructed using a lumped system, such as those applied by Haas [24] or Lee *et al*. [25], but not a split system, such as those adopted in the MEST-C score. Previous studies demonstrated very good reproducibility of the HG and MEST-C scoring systems [8, 11, 33]. We and others observed a significant association between the levels of HG and kidney outcomes [8, 16]. In contrast, a review comprising 19 retrospective studies including >7000 patients correlated MEST parameters with kidney outcomes. Several studies showed significant relationships between the M [*n* = 5 (26.3%)], E [*n* = 4 (21.1%)], S [*n* = 7 (36.8%)] and T [*n* =13 (68.4%)] parameters and poor kidney outcomes [26]. Therefore, there appear to be some predictive advantages for prognosis in each HG and MEST-C score. There is a current J-IGACS cohort effort to compare the prognostic performance of the two grading systems.

CG is a simple risk stratification method that uses proteinuria and eGFR [15], identified as strong predictors of kidney survival by many previous studies [9, 27–29]. Although the discriminative performance of CG for kidney survival was not as good as the RF-RG or HG, CG was significantly associated with kidney survival and ProR.

The RF-RG is a simple, visually presented risk stratification consisting of four levels and can be practically used in clinical settings. The mobile International Risk Prediction Tool for IgAN was proposed in 2019, using available clinical and laboratory risk factors (eGFR, blood pressure, proteinuria, etc.) and the MEST-C scoring system [3]. The univariate RF-RG model and International Risk Prediction Tool had similar (good) discrimination ability, as evidenced by Harrell's C-statistics of 0.81–0.82. A Chinese validation study found the International Risk Prediction Tool to have good discriminative capacity [30]. However, a recent study from Korea showed lower prediction ability of the International Risk Prediction Tool using baseline characteristics, with an AUC of 0.69 (not good) [31]. Our patients’ baseline characteristics differed considerably from the European cohort [32]. Our cohort showed a lower UPER, less frequent RASi use and more frequent corticosteroid therapy and tonsillectomy. Using an International Risk Prediction Tool for kidney prognosis in patients with IgAN in East Asia is controversial. Since East Asia is a high-risk IgAN region genetically [11] and many cases are diagnosed with relatively mild conditions, models that reflect East Asian patients’ characteristics are needed.

To evaluate the validity of general therapeutic goals for the medical treatment of IgAN, we used kidney survival and remission of urinary abnormality as targeted outcomes. Remission of urinary abnormalities during follow-up only occurred in patients with urinary abnormalities at the time of kidney biopsy. We examined patients with both proteinuria and haematuria at baseline, as dataset 4 analysed the relationship between urinary abnormality remission and primary outcomes [19].

In the multivariate model including the RF-RG, we observed remarkable improvement in the AIC by including urinary abnormalities (Table 5). To the best of our knowledge, this is the first study to find that ProR and HemR during follow-up is independently associated with favourable kidney outcomes. This is also the first study to determine that clinicopathological risk stratification can predict urinary protein remission, increasingly recognized as a favourable surrogate kidney outcome [13, 33, 34].

Our study had several limitations. First, the follow-up period was relatively short. This resulted in relatively few kidney outcomes, especially in patients with mild and moderate clinical risk factors. Second, our results were derived from Japanese patients, who demonstrate the highest susceptibility to IgAN globally. Because of this, our findings may not be generalized to patients with IgAN from other geographic regions. Thus our results should be confirmed in different ethnicities and countries.

Our study also features several strengths. We enrolled patients with IgAN with a wide range of characteristics. Then, we evaluated all clinical and analytic parameters of interest (Table 2). We found that the RF-RG—determined by combining HG and CG levels—did not demonstrate superior discrimination ability to HG alone. Within the context of the present grading system, better prognostic tools may be achievable by re-evaluating proteinuria or eGFR thresholds in CG.

## CONCLUSIONS

This nationwide prospective cohort study found that the RF-RG independently associated with ProR and kidney survival. The RF-RG may have satisfactory clinical utility in patients with IgAN since it has good predictive ability for those outcomes.

## Supplementary Material

sfad294_Supplemental_File

## Data Availability

The data underlying this article will be shared upon reasonable request to the corresponding author.
