# Supplementary material for: Clinicopathological prognostic stratification for proteinuria and kidney survival in IgA nephropathy: a Japanese prospective cohort study
Source: Clin Kidney J. 2023 Nov 27;17(1):sfad294. doi: 10.1093/ckj/sfad294 (PMC10783253; doi:10.1093/ckj/sfad294)

**Table of Contents**

**Supplemental Table**

**Table S1. J-IGACS Investigators**

**Table S2. Baseline characteristics and follow-up period according to CG and HG in enrolled 991 patients in Dataset1**

**Table S3. Details of univariate and multivariate Cox regression models for primary outcome**

**Table S4. Univariate and multivariate Cox regression models for a decrease in eGFR from the baseline or dialysis induction**

**Table S5. Univariate and multivariate Fine-Gray regression models for proteinuria remission**

**Table S6. Univariate and multivariate Fine-Gray regression models for hematuria remission**

**Table S7. Details of univariate and multivariate Cox regression models for primary outcome including CG or HG**

**Table S8. Univariate and multivariate Fine-Gray regression models including CG or HG for proteinuria remission**

**Table S9. Univariate and multivariate Fine-Gray regression models including CG or HG for hematuria remission**

**Table S10. Baseline characteristics and follow-up period in 635 patients with both proteinuria and hematuria at baseline (Dataset 4)**

**_**

**Supplemental Figure**

**Figure S1. Comparing primary outcomes among CGI-III and HG I-IV.**

**Figure S2.** **Comparison of proteinuria remission among CG I-III and HG I-IV.**

**Figure S3. Comparison of hematuria remission among CG I-III and HG I-IV.**

| **Table S1. J-IGACS Investigator affiliations** |
| --- |
| Institutions, administrative divisions |
| Asahikawa Medical University Hospital, Hokkaido |
| Japan Community Health Care Organization Sendai Hospital, Miyagi |
| Yamagata University Hospital, Yamagata |
| Gunma University Hospital, Gunma |
| Jichi Medical University Hospital, Tochigi |
| Japanese Red Cross Ashikaga Hospital, Tochigi |
| University of Tsukuba Hospital, Ibaraki |
| Tokyo Medical University Ibaraki Medical Center, Ibaraki |
| The Jikei University School of Medicine Kashiwa Hospital, Chiba |
| Dokkyo Medical University Saitama Medical Center, Saitama |
| Kawaguchi Municipal Medical Center, Saitama |
| Tokyo Women's Medical University Hospital, Tokyo |
| Tokyo Women's Medical University Hospital, Pediatrics, Tokyo |
| Juntendo University Hospital, Tokyo |
| The Jikei University School of Medicine Hospital, Tokyo |
| The Jikei University School of Medicine Katsushika Medical Center, Tokyo |
| The Jikei University School of Medicine Daisan Hospital, Tokyo |
| Nippon Medical School Hospital, Tokyo |
| Showa University Hospital, Tokyo |
| Teikyo University Hospital, Tokyo |
| Tokyo Metropolitan Children's Medical Center, Tokyo |
| St. Marianna University School of Medicine Hospital, Kanagawa |
| Tokai University Hospital, Kanagawa |
| Niigata University Medical & Dental Hospital, Niigata |
| Nagoya University Hospital, Aichi |
| Fujita Health University Hospital, Aichi |
| Kanazawa University Hospital, Ishikawa |
| National Hospital Organization Kanazawa Medical Center, Ishikawa |
| Kyoto University Hospital, Kyoto |
| Osaka City University Hospital, Osaka |
| Kitano Hospital, Osaka |
| National Hospital Organization Osaka Medical Center, Osaka |
| Toyonaka Municipal Hospital, Osaka |
| Kobe University Hospital, Hyougo |
| Shimane University Hospital, Shimane |
| Tottori University Hospital, Tottori |
| National Hospital Organization Kyushu Medical Center, Fukuoka |
| National Hospital Organization Fukuokahigashi Medical Center, Fukuoka |
| Japanese Red Cross Fukuoka Hospital, Fukuoka |
| Fukuoka University Hospital, Fukuoka |
| Kyushu University Hospital, Fukuoka |
| Miyazaki University Hospital, Miyazaki |
| Nagasaki University Hospital, Nagasaki |
| Tokushima University Hospital, Tokushima |

| **Table S2. Baseline characteristics and follow-up period according to CG and HG in enrolled 991 patients in Dataset1** | | | | | | | | |
| --- | --- | --- | --- | --- | --- | --- | --- | --- |
|  | Overall (N=991) | Clinicopathological stratification | | | | | | |
|  |  | CG | | | HG | | | |
|  |  | Ⅰ | Ⅱ | Ⅲ | Ⅰ | Ⅱ | Ⅲ | Ⅳ |
|  |  | (N=432) | (N=338) | (N=221) | (N=620) | (N=245) | (N=90) | (N=36) |
| ***At baseline*** |  |  |  |  |  |  |  |  |
| Age, median (IQR), years | 37.1  (26.8-50.4) | 35.9 (23.6-46.3) | 33.6 (25.3-43.4) | 51.1 (40.7-62.7) | 33.1  (23.6-45.6) | 43.6 (32.4-56.7) | 43.6 (34.7-61.7) | 46.2 (30.6-54.4) |
| Women, number (%) | 502 (50.7) | 231 (53.5) | 176 (52.0) | 95 (43.0) | 314 (50.6) | 129 (52.7) | 44 (48.9) | 15 (41.7) |
| MAP, mean±SD, mmHg | 90.1±13.5 | 86.6±11.8 | 89.5±13.2 | 98.1±13.7 | 87.8±12.6 | 91.3±12.9 | 98.4±14.4 | 101.1±16.1 |
| eGFR, mean±SD, ml/min/1.73 m^2^ | 75.4±28.7 | 83.5±25.3 | 87.5±21.8 | 41.0±14.0 | 85.4±25.3 | 66.1±23.9 | 48.5±24.0 | 32.9±23.7 |
| UprR, median (IQR), g/day | 0.58 (0.28-1.18) | 0.24 (0.11-0.36) | 0.92 (0.65-1.44) | 1.37 (0.89-2.48) | 0.42 (0.20-0.80) | 0.84 (0.47-1.46) | 1.39 (0.92-2.94) | 2.27 (0.91-4.03) |
| U-RBC, >20 /HPF, number  (%) | 514 (51.9) | 217 (50.2) | 154 (51.0) | 107 (53.2) | 331 (53.4) | 127 (51.8) | 47 (52.2) | 9 (25.0) |
| UA, mean±SD, mg/dl | 5.9±1.6 | 5.5±1.5 | 5.5±1.4 | 7.1±1.6 | 5.5±1.5 | 6.1±1.6 | 6.9±1.7 | 7.7±1.5 |
| RASi, number (%) | 567 (57.2) | 181 (41.9) | 204 (60.4) | 182 (82.4) | 282 (45.5) | 170 (69.4) | 82 (91.1) | 33 (91.7) |
| CS0/CS1/CS2, number (%) | 357/57/577 | 207/20/205 | 75/19/244 | 75/18/128 | 241/33/346 | 71/16/158 | 27/7/56 | 18/1/17 |
|  | (36.0/5.8/58.2) | (47.9/4.6/47.5) | (22.2/5.6/72.2) | (33.9/8.1/57.9) | (38.9/5.3/55.8) | (29.0/6.5/64.5) | (30.0/7.8/62.2) | (50.0/2.8/47.2) |
| Tx, number (%) | 425 (42.9) | 183 (42.4) | 167 (49.4) | 75 (33.9) | 271 (43.7) | 110 (44.9) | 38 (42.2) | 6 (16.7) |
| ***Follow up*** |  |  |  |  |  |  |  |  |
| Follow up period, median (IQR) , months | 66 (30-90) | 60 (24-90) | 72 (36-96) | 60 (24-90) | 66 (30-90) | 78 (36-96) | 60 (24-90) | 24 (12-42) |
| **Abbreviations;** CG clinical grade, HG histological grade, MAP mean arterial pressure, eGFR estimated glomerular filtration rate, UprR urinary protein excretion rate, U-RBC urine sediment of red blood cell, HPF high power field, UA uric acid, RASi renin angiotensine system inhibitor, CS0 no corticosteroid therapy, CS1 oral corticosteroid therapy without pulse regimen, CS2 pulse corticosteroid therapy, Tx tonsillectomy, IQR interquartile range. | | | | | | | | |

| **Table S3.** Details of univariate and multivariate Cox regression models for primary outcome | | | | | | | |  |  |  |  | |  |
| --- | --- | --- | --- | --- | --- | --- | --- | --- | --- | --- | --- | --- | --- |
| Predictors | | Univariate | | | | | Multivariate | | | | | |  |
|  |  |  |  |  |  |  |  |  |  |  |  |  |  |
|  |  |  |  |  |  |  |  |  |  |  |  |  |  |
|  |  |  |  |  |  |  |  |  |  |  |  |  |  |
|  |  |  |  |  |  |  |  |  |  |  |  | |  |
|  |  | HR (95%CI) | P value | AIC | C-statistics | | HR (95%CI) | P value | AIC | C-statistics | | |  |
|  |  |  |  |  | Harrell's | Uno's |  |  |  | Harrell's | | Uno's |  |
| ***Clinicopathological stratification*** | |  |  |  |  |  |  |  |  |  | |  |  |
| RF-RG | I | reference | <0.001 | 992.2 | 0.81 (0.76-0.86) | 0.73 (0.64-0.82) | reference | <0.001 | 959.0 | 0.85 (0.80-0.91) | | 0.80 (0.73-0.87) |  |
|  | II | 2.78 (1.12-6.93) |  |  |  |  | 3.20 (1.26-8.14) |  |  |  |  |  |  |
|  | III | 7.15 (2.90-17.6) |  |  |  |  | 5.01 (1.79-14.0) |  |  |  |  |  |  |
|  | IV | 33.4 (14.1-79.0) |  |  |  |  | 15.2 (5.10-45.3) |  |  |  |  |  |  |
| ***Clinical factors*** | |  |  |  |  |  |  |  |  |  |  |  |  |
| Age, per 10 year | | 1.51 (1.32-1.73) | <0.001 | 1072.8 | 0.69 | 0.65 | 1.05 (0.88-1.25) | 0.748 |  |  |  |  |  |
| Female versus Male | | 0.69 (0.45-1.06) | 0.087 | 1106.0 | 0.54 | 0.55 | 1.00 (0.62-1.25) | 0.987 |  |  |  |  |  |
| MAP, per 10mmHg | | 1.47 (1.28-1.68) | <0.001 | 1081.1 | 0.67 | 0.66 | 1.10 (0.93-1.29) | 0.487 |  |  |  |  |  |
| eGFR, per 10ml/min | | 0.61 (0.55-0.68) | <0.001 | 1107.2 | 0.80 | 0.71 | 0.87 (0.75-1.01) | 0.086 |  |  |  |  |  |
| UprR, per 1.0g/day | | 1.09 (1.05-1.12) | <0.001 | 1094.7 | 0.74 | 0.71 | 1.08 (1.03-1.13) | 0.008 |  |  |  |  |  |
| U-RBC>20/HPF, Yes versus No | | 0.50 (0.33-0.78) | 0.002 | 1099.0 | 0.61 | 0.59 | 0.76 (0.47-1.22) | 0.159 |  |  |  |  |  |
| UA, per 1mg/dl | | 1.52 (1.35-1.70) | <0.001 | 1065.5 | 0.67 | 0.66 | 1.07 (0.89-1.28) | 0.512 |  |  |  |  |  |
| **Abbreviations;** RF**-**RG renal failure disease risk group, AIC Akaike's information criterion, HR hazard ratio, CI confidence interval, MAP mean arterial pressure, eGFR estimated glomerular filtration rate, UA uric acid, UprR urinary protein excretion rate, U-RBC urine sediment of red blood cell, HPF high power field, UA uric acid, ND not determined. All multivariate models were adjusted with initial treatment with RASi, glucocorticoid and tonsillectomy in addition to the parameters listed in each column. | | | | | | | | | | | | |  |

| **Table S4.** Univariate and multivariate Cox regression models for a decrease in eGFR from the baseline or dialysis induction | | | | | | | | | |  |
| --- | --- | --- | --- | --- | --- | --- | --- | --- | --- | --- |
| Outcome | | 30% decrease in eGFR | | | | 50% decrease in eGFR | | | |  |
| Predictors | | Univariate | | Multivariate | | Univariate | | Multivariate | |  |
|  |  |  |  |  |  |  |  |  |  |  |
|  |  | HR (95%CI) | P value | HR (95%CI) | P value | HR (95%CI) | P value | HR (95%CI) | P value |  |
|  |  |  |  |  |  |  |  |  |  |  |
| ***Clinicopathological stratification*** | |  |  |  |  |  |  |  |  |  |
| RF-RG | I | reference | <0.001 | reference | <0.001 | reference | <0.001 | reference | 0.021 |  |
|  | II | 3.51 (1.69-7.27) |  | 4.00 (1.89-8.46) |  | 7.30 (0.93-57.6) |  | 11.6 (1.38-88.8) |  |  |
|  | III | 6.57 (3.12-13.8) |  | 6.80 (2.89-16.0) |  | 62.9 (2.85-168.5) |  | 7.93 (0.98-64.1) |  |  |
|  | IV | 21.2 (10.3-43.8) |  | 19.5 (7.78-49.0) |  | 153.8 (21.0-1123.9) |  | 17.4 (2.13-141.9) |  |  |
| ***Clinical factors*** | |  |  |  |  |  |  |  |  |  |
| Age, per 10 year | | 1.47 (1.30-1.65) | <0.001 | 1.16 (1.00-1.36) | 0.057 | 1.71 (1.43-2.04) | <0.001 | 0.92 (7.43-1.15) | 0.471 |  |
| Female versus Male | | 0.72 (0.50-1.04) | 0.082 | 1.06 (0.69-1.63) | 0.791 | 0.50 (0.29-0.88) | 0.015 | 0.96 (0.53-1.74) | 0.471 |  |
| MAP, per 10mmHg | | 1.35 (1.20-1.53) | <0.001 | 1.00 (0.86-1.16) | 0.996 | 1.57 (1.33-1.85) | <0.001 | 1.01 (0.82-1.25) | 0.893 |  |
| eGFR, per 10ml/min | | 0.74 (0.68-0.80) | <0.001 | 1.09 (0.96-1.25) | 0.191 | 0.41 (0.35-0.49) | <0.001 | 0.55 (0.43-0.70) | <0.001 |  |
| UprR, per 1.0g/day | | 1.08 (1.04-1.11) | <0.001 | 1.05 (1.00-1.10) | 0.036 | 1.08 (1.03-1.12) | 0.001 | 0.99 (9.10-1.09) | 0.957 |  |
| U-RBC>20/HPF, Yes versus No | | 0.57 (0.39-0.83) | 0.004 | 0.78 (0.52-1.16) | 0.218 | 0.35 (0.20-0.63) | <0.001 | 0.59 (0.31-1.14) | 0.119 |  |
| UA, per 1mg/dl | | 1.41 (1.26-1.57) | <0.001 | 1.11 (0.95-1.29) | 0.189 | 1.83 (1.61-2.07) | <0.001 | 1.26 (1.05-1.51) | 0.014 |  |
| **Abbreviations;** RF-RG renal failuer risk group, HR hazard ratio, CI confidence interval, MAP mean arterial pressure, eGFR estimated glomerular filtration rate, UA uric acid, UprR urinary protein excretion rate, U-RBC urine sediment of red blood cell, HPF high power field, UA uric acid, ND not determined. All multivariate models were adjusted with initial treatment with RASi, glucocorticoid and tonsillectomy in addition to the parameters listed in each column. | | | | | | | | | |  |

| **Table S5.** Univariate and multivariate Fine-Gray regression models for proteinuria remission | | | | | | | |  |
| --- | --- | --- | --- | --- | --- | --- | --- | --- |
| Predictors | | Univariate | | | Multivariate | | |  |
|  |  |  |  |  |  |  |  |  |
|  |  |  |  |  |  |  |  |  |
|  |  | sHR (95%CI) | P value | AIC | sHR (95%CI) | P value | AIC |  |
| ***Clinicopathological stratification*** | |  |  |  |  |  | 6222.0 |  |
| RF-RG | I | reference | <0.001 | 6262.5 | reference | <0.001 |  |  |
|  | II | 0.79 (0.67-0.94) |  |  | 0.75 (0.63-0.91) |  |  |  |
|  | III | 0.52 (0.41-0.66) |  |  | 0.58 (0.44-0.76) |  |  |  |
|  | IV | 0.15 (0.09-0.23) |  |  | 0.19 (0.11-0.33) |  |  |  |
| ***Clinical parameters*** | |  |  |  |  |  |  |  |
| Age, per 10 year | | 0.85 (0.80-0.89) | <0.001 | 6332.1 | 0.99 (0.92-1.06) | 0.775 |  |  |
| Female versus Male | | 1.11 (0.95-1.29) | 0.185 | 6364.7 | 0.95 (0.79-1.13) | 0.553 |  |  |
| MAP, per 10mmHg | | 0.86 (0.81-0.91) | <0.001 | 6345.3 | 1.02 (0.95-1.09) | 0.558 |  |  |
| eGFR, per 10ml/min | | 1.13 (1.11-1.16) | <0.001 | 6297.2 | 1.02 (0.98-1.07) | 0.302 |  |  |
| UprR, per 1.0g/day | | 0.86 (0.76-0.98) | 0.021 | 6343.6 | 0.95 (0.87-1.03) | 0.215 |  |  |
| U-RBC>20/HPF, Yes versus No | | 1.46 (1.25-1.70) | <0.001 | 6348.3 | 1.32 (1.14-1.54) | <0.001 |  |  |
| UA, per 1mg/dl | | 0.87 (0.83-0.91) | <0.001 | 6338.3 | 0.99 (0.93-1.05) | 0.470 |  |  |
| **Abbreviations;** RF**-**RG renal failure disease risk group, sHR subdistribution hazard ratio, AIC Akaike's information criterion, CI confidence interval, MAP mean arterial pressure, eGFR estimated glomerular filtration rate, UprR urinary protein excretion rate, U-RBC urine sediment of red blood cell, HPF high power field, UA uric acid, ND not determined. All multivariate models were adjusted with initial treatment with RASi, glucocorticoid and tonsillectomy in addition to the parameters listed in each column. | | | | | | | |  |

| **Table S6.** Univariate and multivariate Fine-Gray regression models for hematuria remission | | | | | | |  |
| --- | --- | --- | --- | --- | --- | --- | --- |
| Predictors | | Univariate | | | Multivariate | |  |
|  |  | sHR (95%CI) | P value | AIC | sHR (95%CI) | P value | AIC |
| ***Clinicopathological stratification*** | |  |  |  |  | |  |
| RF-RG | I | reference | 0.025 | 7934.2 | reference | 0.026 | 7869.9 |
|  | II | 1.11 (0.95-1.29) |  |  | 0.94 (0.79-1.12) |  |  |
|  | III | 1.11 (0.90-1.37) |  |  | 1.06 (0.83-1.36) |  |  |
|  | IV | 0.68 (0.49-0.94) |  |  | 0.61 (0.41-0.90) |  |  |
| ***Clinical parameters*** | |  |  |  |  |  |  |
| Age, per 10 year | | 0.96 (0.92-1.00) | 0.042 | 7937.6 | 0.99 (0.93-1.06) | 0.846 |  |
| Female versus Male | | 1.13 (0.98-1.29) | 0.094 | 7938.8 | 1.02 (0.87-1.21) | 0.780 |  |
| MAP, per 10mmHg | | 0.96 (0.91-1.02) | 0.158 | 7939.2 | 1.02 (0.96-1.10) | 0.409 |  |
| eGFR, per 10ml/min | | 1.02 (1.00-1.05) | 0.076 | 7938.4 | 0.99 (0.94-1.03) | 0.529 |  |
| UprR, per 1.0g/day | | 1.00 (0.97-1.04) | 0.908 | 7941.0 | 1.01 (0.98-1.05) | 0.563 |  |
| U-RBC>20/HPF, Yes versus No | | 0.78 (0.67-0.90) | <0.001 | 7931.0 | 0.70 (0.61-0.81) | <0.001 |  |
| UA, per 1mg/dl | | 0.96 (0.92-1.00) | 0.046 | 7937.5 | 0.99 (0.93-1.05) | 0.736 |  |
| **Abbreviations;** RF**-**RG renal failure disease risk group, sHR subdistribution hazard ration, AIC Akaike's information criterion, CI confidence interval, MAP mean arterial pressure, eGFR estimated glomerular filtration rate, UprR urinary protein excretion rate, U-RBC urine sediment of red blood cell, HPF high power field, UA uric acid, ND not determined. All multivariate models were adjusted with initial treatment with RASi, glucocorticoid and tonsillectomy in addition to the parameters listed in each column. | | | | | | |  |
|  |  |  |  |  |  |  |  |
|  |  |  |  |  |  |  |  |

| **Table S7.** Details of univariate and multivariate Cox regression models for primary outcome including CG or HG | | | | | | | | | | | | |
| --- | --- | --- | --- | --- | --- | --- | --- | --- | --- | --- | --- | --- |
| Predictors | | Univariate | | | | | Multivariate | | | | | |
|  |  |  |  |  |  |  | Model CG | | | Model HG | | |
|  |  |  |  |  |  |  | AIC | | 971.3 | AIC | | 943.3 |
|  |  |  |  |  |  |  | C-statistics | Harrell's | 0.84 (0.78-0.90) | C-statistics | Harrell's | 0.87 (0.82-0.92) |
|  |  |  |  |  |  |  |  | Uno's | 0.80 (0.73-0.86) |  | Uno's | 0.82 (0.75-0.89) |
|  |  | HR (95%CI) | P value | AIC | C-statistics | | HR (95%CI) | | P value | HR (95%CI) | | P value |
|  |  |  |  |  | Harrell's | Uno's |  |  |  |  |  |  |
| ***Clinicopathological stratification*** | |  |  |  |  |  |  |  |  |  |  |  |
| CG | I | reference | <0.001 | 1028.9 | 0.75 (0.70-0.81) | 0.71 (0.62-0.80) | reference | | <0.001 | ND | | |
|  | II | 2.11 (1.01-4.39) |  |  |  |  | 3.55 (1.61-7.83) | |  |  |  |  |
|  | III | 10.6 (5.53-20.2) |  |  |  |  | 3.91 (1.78-8.56) | |  |  |  |  |
| HG | I | reference | <0.001 | 983.1 | 0.82 (0.77-0.87) | 0.72 (0.64-0.80) | ND | | | reference | | <0.001 |
|  | II | 4.24 (2.27-7.95) |  |  |  |  |  |  |  | 3.50 (1.81-6.77) | |  |
|  | III | 11.2 (5.88-21.4) |  |  |  |  |  |  |  | 6.66 (3.11-14.3) | |  |
|  | IV | 51.7 (26.1-102.6) |  |  |  |  |  |  |  | 18.7 (7.98-44.0) | |  |
| ***Clinical parameters*** | | Refer to Table S3. Same results as in the univariate analysis in Table S3. | | | | |  |  |  |  |  |  |
| Age, per 10 year | |  |  |  |  |  | 0.89 (0.75-1.06) | | 0.195 | 1.13 (0.93-1.38) | | 0.624 |
| Female versus Male | |  |  |  |  |  | 1.00 (0.62-1.63) | | 0.994 | 0.96 (0.59-1.57) | | 0.877 |
| MAP, per 10mmHg | |  |  |  |  |  | 1.07 (0.91-1.26) | | 0.403 | 1.11 (0.91-1.36) | | 0.279 |
| eGFR, per 10ml/min | |  |  |  |  |  | 0.70 (0.59-0.84) | | <0.001 | 0.93 (0.79-1.09) | | 0.07 |
| UprR, per 1.0g/day | |  |  |  |  |  | 1.06 (1.01-1.10) | | 0.020 | 1.09 (1.04-1.15) | | 0.001 |
| U-RBC>20/HPF, Yes versus No | |  |  |  |  |  | 0.63 (0.40-1.01) | | 0.056 | 0.61 (0.34-1.07) | | 0.255 |
| UA, per 1mg/dl | |  |  |  |  |  | 1.08 (0.91-1.28) | | 0.361 | 0.97 (0.80-1.18) | | 0.462 |
| **Abbreviations;** CG clinical grade, HG histological grade, AIC Akaike's information criterion, HR hazard ratio, CI confidence interval, MAP mean arterial pressure, eGFR estimated glomerular filtration rate, UA uric acid, UprR urinary protein excretion rate, U-RBC urine sediment of red blood cell, HPF high power field, UA uric acid, ND not determined. All multivariate models were adjusted with initial treatment with RASi, glucocorticoid and tonsillectomy in addition to the parameters listed in each column. | | | | | | | | | | | | |

| **Table S8.** Univariate and multivariate Fine-Gray regression models including CG or HG for proteinuria remission | | | | | | | | | | |
| --- | --- | --- | --- | --- | --- | --- | --- | --- | --- | --- |
| Predictors | | Univariate | | | Multivariate | | | | | |
|  |  |  |  |  | Model CG for ProR | | | Model HG for ProR | | |
|  |  | sHR (95%CI) | P value | AIC | sHR (95%CI) | P value | AIC | sHR (95%CI) | P value | AIC |
| ***Clinicopathological stratification*** | |  |  |  |  |  | 6242.2 |  |  | 6215.1 |
| CG | I | reference | <0.001 | 6300.0 | reference | <0.001 |  | ND | |  |
|  | II | 0.78 (0.67-0.92) |  |  | 0.70 (0.58-0.85) |  |  |  |  |  |
|  | III | 0.37 (0.29-0.47) |  |  | 0.51 (0.38-0.69) |  |  |  |  |  |
| HG | I | reference | <0.001 | 6263.7 | ND | |  | reference | <0.001 |  |
|  | II | 0.64 (0.53-0.77) |  |  |  |  |  | 0.67 (0.56-0.81) |  |  |
|  | III | 0.30 (0.22-0.42) |  |  |  |  |  | 0.37 (0.26-0.52) |  |  |
|  | IV | 0.09 (0.03-0.25) |  |  |  |  |  | 0.15 (0.05-0.42) |  |  |
| ***Clinical parameters*** | |  |  |  |  |  |  |  |  |  |
| Age, per 10 year | | Refer to Table S4. Same results as in the univariate analysis in Table S4. | | | 1.02 (0.95-1.09) | 0.669 |  | 0.98 (0.92-1.05) | 0.625 |  |
| Female versus Male | |  |  |  | 0.88 (0.74-1.05) | 0.164 |  | 0.97 (0.81-1.16) | 0.740 |  |
| MAP, per 10mmHg | |  |  |  | 1.02 (0.95-1.09) | 0.671 |  | 1.00 (0.94-1.07) | 0.995 |  |
| eGFR, per 10ml/min | |  |  |  | 1.06 (1.02-1.11) | 0.008 |  | 1.03 (0.99-1.07) | 0.145 |  |
| UprR, per 1.0g/day | |  |  |  | 0.94 (0.84-1.04) | 0.194 |  | 0.93 (0.85-1.02) | 0.121 |  |
| U-RBC>20/HPF, Yes versus No | |  |  |  | 1.34 (1.15-1.56) | <0.001 |  | 1.31 (1.13-1.52) | <0.001 |  |
| UA, per 1mg/dl | |  |  |  | 0.96 (0.90-1.02) | 0.201 |  | 0.97 (0.91-1.04) | 0.417 |  |
| **Abbreviations;** CG clinical grade, HG histological grade, ProR proteinuria remission, sHR subdistribution hazard ratio, AIC Akaike's information criterion, CI confidence interval, MAP mean arterial pressure, eGFR estimated glomerular filtration rate, UprR urinary protein excretion rate, U-RBC urine sediment of red blood cell, HPF high power field, UA uric acid, ND not determined. All multivariate models were adjusted with initial treatment with RASi, glucocorticoid and tonsillectomy in addition to the parameters listed in each column. | | | | | | | | | | |

| **Table S9.** Univariate and multivariate Fine-Gray regression models including CG or HG for hematuria remission | | | | | | | | |  |
| --- | --- | --- | --- | --- | --- | --- | --- | --- | --- |
| Predictors | | Univariate | | | Multivariate | | | |  |
|  |  |  |  |  | Model CG for HemR | | Model HG for HemR | |  |
|  |  |  |  |  | AIC | 7876.7 | AIC | 7869.6 |  |
|  |  | sHR (95%CI) | P value | AIC | sHR (95%CI) | P value | sHR (95%CI) | P value |  |
| ***Clinicopathological stratification*** | |  |  |  |  |  |  |  |  |
| CG | I | reference | 0.283 | 7940.6 | reference | 0.489 | ND | |  |
|  | II | 1.08 (0.93-1.25) |  |  | 0.90 (0.75-1.08) |  |  |  |  |
|  | III | 0.91 (0.74-1.11) |  |  | 0.91 (0.70-1.18) |  |  |  |  |
| HG | I | reference | 0.024 | 7932.7 | ND | | reference | 0.030 |  |
|  | II | 1.05 (0.88-1.24) |  |  |  |  | 0.98 (0.82-1.18) |  |  |
|  | III | 0.89 (0.66-1.14) |  |  |  |  | 0.80 (0.59-1.07) |  |  |
|  | IV | 0.44 (0.24-0.78) |  |  |  |  | 0.42 (0.23-0.78) |  |  |
| ***Clinical parameters*** | |  |  |  |  |  |  |  |  |
| Age, per 10 year | | Refer to Table S5. Same results as in the univariate analysis in Table S5. | | | 1.01 (0.94-1.08) | 0.852 | 0.99 (0.93-1.06) | 0.820 |  |
| Female versus Male | |  |  |  | 1.01 (0.85-1.19) | 0.936 | 1.04 (0.88-1.24) | 0.636 |  |
| MAP, per 10mmHg | |  |  |  | 1.02 (0.96-1.09) | 0.521 | 1.02 (0.96-1.09) | 0.514 |  |
| eGFR, per 10ml/min | |  |  |  | 1.00 (0.96-1.05) | 1.000 | 0.98 (0.94-1.03) | 0.435 |  |
| UprR, per 1.0g/day | |  |  |  | 1.01 (0.97-1.05) | 0.696 | 1.01 (0.98-1.05) | 0.393 |  |
| U-RBC>20/HPF, Yes versus No | |  |  |  | 0.72 (0.62-0.83) | <0.001 | 0.70 (0.60-0.81) | <0.001 |  |
| UA, per 1mg/dl | |  |  |  | 0.98 (0.92-1.05) | 0.550 | 0.99 (0.93-1.03) | 0.808 |  |
| **Abbreviations;** CG clinical grade, HG histological grade, HemR hematusia remission, sHR subdistribution hazard ration, AIC Akaike's information criterion, CI confidence interval, MAP mean arterial pressure, eGFR estimated glomerular filtration rate, UprR urinary protein excretion rate, U-RBC urine sediment of red blood cell, HPF high power field, UA uric acid, ND not determined. All multivariate models were adjusted with initial treatment with RASi, glucocorticoid and tonsillectomy in addition to the parameters listed in each column. | | | | | | | | |  |
|  |  |  |  |  |  |  |  |  |  |
|  |  |  |  |  |  |  |  |  |  |

| **Table S10. Baseline characteristics and follow-up period in 635 patients with both proteinuria and hematuria at baseline (Dataset 4)** | | | | | |
| --- | --- | --- | --- | --- | --- |
|  | Overall (N=635) | RF-RG | | | |
|  |  | Ⅰ | Ⅱ | Ⅲ | Ⅳ |
|  |  | (N=142) | (N=284) | (N=138) | (N=71) |
| **At baseline** |  |  |  |  |  |
| Age, median (IQR), years | 37.7  (27.7-50.3) | 32.8 (21.5-41.5) | 34.8 (25.7-45.1) | 47.9 (38.0-61.0) | 47.1 (35.8-63.3) |
| Women, number (%) | 315 (49.6) | 70 (49.3) | 148 (52.1) | 67 (48.6) | 30 (42.3) |
| MAP, mean±SD, mmHg | 90.5±13.9 | 84.4±11.6 | 87.0±13.3 | 95.3±12.3 | 100.4±14.2 |
| eGFR, mean±SD, ml/min/1.73 m^2^ | 73.6±29.5 | 89.1±23.2 | 81.4±24.2 | 52.9±17.9 | 33.6±14.3 |
| UprR, median (IQR), g/day | 0.78 (0.44-1.43) | 0.34 (0.27-0.43) | 0.84 (0.57-1.25) | 1.33 (0.73-2.07) | 2.13 (1.13-3.91) |
| U-RBC, >20 /HPF, number  (%) | 358 (56.4) | 84 (59.2) | 153 (53.9) | 86 (62.3) | 35 (49.3) |
| UA, mean±SD, mg/dl | 6.0±1.6 | 5.5±1.6 | 5.5±1.4 | 6.7±1.4 | 7.5±1.7 |
| RASi, number (%) | 398 (62.7) | 50 (35.2) | 168 (59.2) | 116 (84.1) | 64 (90.1) |
| CS0/CS1/CS2, number (%) | 188/43/404 | 66/7/69 | 63/19/202 | 36/10/92 | 23/7/41 |
|  | (29.6/6.8/63.6) | (46.5/4.9/48.6) | (22.2/6.7/71.1) | (26.1/7.2/66.7) | (32.4/9.9/57.7) |
| Tx, number (%) | 287 (45.2) | 67 (47.2) | 140 (49.3) | 55 (39.9) | 25 (35.2) |
| **Follow up** |  |  |  |  |  |
| Follow up period, median (IQR) , months | 54 (30-84) | 54 (30-84) | 60 (36-84) | 60 (30-84) | 24 (12-48) |
| **Abbreviations;** RF-RG renal failure risk group, MAP mean arterial pressure, eGFR estimated glomerular filtration rate, UprR urinary protein excretion rate, U-RBC urine sediment of red blood cell, HPF high power field, UA uric acid, RASi renin angiotensine system inhibitor, CS0 no corticosteroid therapy, CS1 oral corticosteroid therapy without pulse regimen, CS2 pulse corticosteroid therapy, Tx tonsillectomy. IQR interquartile range. | | | | | |

**Figure S1.** **The association between a decrease of eGFR or dialysis induction and RF-RG**.

Kaplan–Meier survival curves showed significantly different kidney outcomes relative to RF-RG level. In an exploratory analysis, 30% (A) and 50% (B) decrease in eGFR from baseline were evaluated as outcomes, with significant differences in both.

**Figure S2. Comparing primary outcomes among CGI-III and HG I-IV.**

Kaplan–Meier survival curves reveal significantly different renal outcomes among CG (A) and HG (B) levels. Univariate (upper panels) and multivariate (lower panels) Cox regression models reveal significant prediction for the primary outcome by CG and HG (C). Multivariate models were adjusted for age, sex, MAP, eGFR, UprR, U-RBC, UA, and initial treatment with RASi, glucocorticoid, and tonsillectomy. All models showed a good fit (i.e., low AIC) and good discrimination (i.e., high C-statistics).

**Figure S3. Comparison of proteinuria remission among CG I-III and HG I-IV, respectively.**

The CIF curves and Gray’s tests show significantly different rates of proteinuria remission among CGI-III (A) and HG I-IV (B). Panel C shows the sub-distribution hazard ratio (sHR) of CG and HG for proteinuria remission according to the Fine-Gray model (see Table S7). Abbreviations: CG, clinical grade; HG, histological grade; sHR, sub-distribution hazard ratio; CI, confidence interval.

**Figure S4. Comparison of hematuria remission among CG I-III and HG I-IV**. The CIF curves show the cumulative rate of hematuria remission is small as levels of CG (A) and HG (B) increase. Gray’s tests show significantly different rates of hematuria remission among HG I-IV (B). Panel C shows the sHR of CG and HG for hematuria remission according to the Fine-Gray model (also see Table S8). Abbreviations: CG, clinical grade; HG, histological grade; sHR, sub-distribution hazard ratio; CI, confidence interval.


Supplement: sfad294_Supplemental_File [file sfad294_supplemental_file.docx]
